# Correction to: CRISPRi screen for enhancing heterologous α-amylase yield in *Bacillus subtilis*

**DOI:** 10.1093/jimb/kuad003

**Published:** 2023-03-20

**This is a correction** to: Adrian Sven Geissler, Annaleigh Ohrt Fehler, Line Dahl Poulsen, Enrique González-Tortuero, Thomas Beuchert Kallehauge, Ferhat Alkan, Christian Anthon, Stefan Ernst Seemann, Michael Dolberg Rasmussen, Anne Breüner, Carsten Hjort, Jeppe Vinther, Jan Gorodkin, CRISPRi screen for enhancing heterologous α-amylase yield in *Bacillus subtilis*, *Journal of Industrial Microbiology and Biotechnology*, kuac028, https://doi.org/10.1093/jimb/kuac028

In the originally published version of this manuscript, there were errors in the addresses for affiliations 1 and 2. These should read: “1 Center for non-coding RNA in Technology and Health, Department of Veterinary and Animal Sciences, University of Copenhagen, 1870 Frederiksberg, Denmark 2 Section for Computational and RNA Biology, Department of Biology, University of Copenhagen, 2200 Copenhagen, Denmark” instead of: “1 Center for non-coding RNA in Technology and Health, Department of Veterinary and Animal Sciences, University of Copenhagen, 1172 Copenhagen, Denmark 2 Section for Computational and RNA Biology, Department of Biology, University of Copenhagen, 1172 Copenhagen, Denmark”.

These errors have been corrected in the article.

